# Engaging with stakeholders to inform the development of a decision-support tool for the NHS health check programme: qualitative study

**DOI:** 10.1186/s12913-020-05268-5

**Published:** 2020-05-11

**Authors:** Lirije Hyseni, Maria Guzman-Castillo, Chris Kypridemos, Brendan Collins, Ellen Schwaller, Simon Capewell, Angela Boland, Rumona Dickson, Martin O’Flaherty, Kay Gallacher, Peter Hale, Ffion Lloyd-Williams

**Affiliations:** 1 Department of Public Health & Policy, University of Liverpool, 3rd floor Whelan Building, Room 3.09, Liverpool, L69 3GB UK; 2 Department of Health Services Research, Liverpool Reviews and Implementation Group, University of Liverpool, Liverpool, UK

**Keywords:** Stakeholder engagement, Co-production, Model development, NHS health checks, Qualitative, Public health

## Abstract

**Background:**

The NHS Health Check Programme is a risk-reduction programme offered to all adults in England aged 40–74 years. Previous studies mainly focused on patient perspectives and programme delivery; however, delivery varies, and costs are substantial. We were therefore working with key stakeholders to develop and co-produce an NHS Health Check Programme modelling tool (workHORSE) for commissioners to quantify local effectiveness, cost-effectiveness, and equity. Here we report on Workshop 1, which specifically aimed to facilitate engagement with stakeholders; develop a shared understanding of current Health Check implementation; identify what is working well, less well, and future hopes; and explore features to include in the tool.

**Methods:**

This qualitative study identified key stakeholders across the UK via networking and snowball techniques. The stakeholders spanned local organisations (NHS commissioners, GPs, and academics), third sector and national organisations (Public Health England and The National Institute for Health and Care Excellence). We used the validated Hovmand “group model building” approach to engage stakeholders in a series of pre-piloted, structured, small group exercises. We then used Framework Analysis to analyse responses.

**Results:**

Fifteen stakeholders participated in workshop 1. Stakeholders identified continued financial and political support for the NHS Health Check Programme. However, many stakeholders highlighted issues concerning lack of data on processes and outcomes, variability in quality of delivery, and suboptimal public engagement. Stakeholders’ hopes included maximising coverage, uptake, and referrals, and producing additional evidence on population health, equity, and economic impacts. Key model suggestions focused on developing good-practice template scenarios, analysis of broader prevention activities at local level, accessible local data, broader economic perspectives, and fit-for-purpose outputs.

**Conclusions:**

A shared understanding of current implementations of the NHS Health Check Programme was developed. Stakeholders demonstrated their commitment to the NHS Health Check Programme whilst highlighting the perceived requirements for enhancing the service and discussed how the modelling tool could be instrumental in this process. These suggestions for improvement informed subsequent workshops and model development.

## Background

Cardiovascular disease (CVD) is the leading cause for mortality and morbidity in the UK, accounting for a quarter of all deaths [1]. The NHS Health Check Programme (NHSHCP) was implemented across England in 2009 with the aim of preventing CVD. The NHS Health Check is offered at five-year intervals to all adults aged 40–74 years who do not have pre-existing vascular conditions. Besides preventing CVD, the programme is designed to also focus on detecting diabetes and chronic kidney disease and raise awareness of dementia. The programme involves CVD risk stratification. People identified as being high-risk are offered appropriate treatment including behavioural change interventions, statins for high cholesterol and antihypertensive drugs for high blood pressure [2]. Since 2013, as part of the Health and Social Care Act, Local Authorities have had the statutory responsibility to commission the programme [3]. Even though the NHSHCP is a national programme, there is substantial variation in its implementation and delivery within and between Local Authorities [4–6]. The programme is delivered predominantly by General Practices (GPs), also by community pharmacies and leisure centres. Outreach pilots have included venues such as pubs and libraries [5]. Currently invitation and attendance data are universally collected as part of the programme. However, other outcome data, such as people diagnosed or treated are not routinely collected.

There is inconsistent evidence regarding the effectiveness, cost-effectiveness and equity impact of the NHSHCP. The programme was implemented based on a 2008 model built for the Department of Health which suggested that NHS Health Checks would have an incremental cost-effectiveness ratio (ICER) of £2480 per quality-adjusted life-year (QALY) gained [7]. With such a low ICER per QALY gained, the NHSHCP would be regarded as being very cost effective. However, the initial estimation of the ICER was arguably based on selected evidence [7]. When health policies are considered or implemented, it is essential to ensure that they provide high-quality and cost-effective programmes and services. Evidence informed decisions are instrumental in this process; however, barriers to using evidence to inform decision-making remain [8]. The policy and programme decision making process is influenced by a range of factors including budget and resource constraints, political environment, and public perceptions [8–11]. Furthermore, research evidence for the prevention of chronic diseases is often complex with many contributing risk factors, making it hard to understand and predict which interventions might best tackle the problem [12, 13]. An ‘evidence-policy gap’ has been identified whereby there is an apparent disconnect between the production of evidence and the use of evidence by policymakers. In order to enhance informed decision-making, it is therefore crucially important to involve policy makers [14]. Dynamic simulation models can be used to synthesise evidence, model potential intervention options and their outcomes and recreate complex systems to help inform decision-making [15, 16]. Freebairn et al. [17] explored the use of end-user decision makers in participatory simulation modelling and reported that the co-production element of the participatory approach was crucial in understanding the modelling process. Further benefits included trust in the model and its outputs and simulating the effect of potential interventions [17].

There is thus a growing awareness within the research community that engaging with stakeholders is vitally important for the development of modelling tools which are relevant, useful and used in real life. Several voices within the modelling community have repeatedly stated the need to access the expertise and knowledge of stakeholders to be able to successfully construct models of strategic problems in policy. The participation of stakeholders in modelling has primarily evolved to what is now formally called group model building (GMB) [18]. GMB is a participatory method for involving people in designing, creating and/or validating models. GMB consists of one or more sessions (workshops) with a carefully selected group of stakeholders and the use of small structured exercises with specific objectives and outputs; and the extensive use of facilitation, discussions and analysis.

This study was part of a two-year project aimed to provide a validated open source / open access, flexible computer modelling tool to enable local commissioners to quantify the potential effectiveness, cost-effectiveness and equity impact of their local NHSHCP, by building on the solid foundation of the existing IMPACT_NCD_ model [19]. This innovative project is engaging with key stakeholders via four, whole-day workshops to co-produce the workHORSE modelling tool for local commissioners to inform desirable features of the user-friendly model and identify additional locally relevant future implementation scenarios. In this paper, we report on the engagement with the stakeholders in workshop 1, where we aimed to explore the NHSHCP in terms of what is working well, less well and future hopes, and explore features to potentially include in a useful decision-support tool for stakeholders. The subsequent three workshops explored: a) modelling potential alternative NHS HC implementations, further features the tool needed to improve on; b) ranking model outputs and visualisations according to importance and usefulness, co-designing realistic model scenarios (previously raised by stakeholders); and c) dissemination and demonstration of the final workHORSE tool, with stakeholders presenting different scenarios.

## Methods

### Study design

We conducted a qualitative study to identify aspects within the NHSHCP that are working well, less well and future hopes, and explore features to include in the workHORSE tool. To ensure proper conduct, we adhered to the SRQR reporting guidelines (Standards for Reporting Qualitative Research) [20].

### Stakeholder recruitment

We developed a stakeholder recruitment grid based on potential stakeholder groups involved in the NHSHCP at the strategic, commissioning, delivery and academic level. We identified 28 potential stakeholder groups (organisations), and used our networks to identify individuals within these groups, which were subsequently added to the grid by the workHORSE project team. The final recruitment grid contained a diverse group of stakeholders from different organisations including Public Health England (national and regional level), British Heart Foundation, Diabetes UK, Alzheimer’s UK, NICE, BMA, Alcohol Research UK, North West Strategic Clinical Network, Director of Public Health, Local Government Association, Clinical Commissioning Groups (CCGs), Local Authorities (LAs), GPs, Pharmacies and Academics. Over 50 stakeholders were sent an email invitation to attend workshop 1. If stakeholders were unable to attend workshop 1, we used snowballing techniques to identify other individuals at their organisation to invite. We had a maximum of twenty spaces available in the workshop.

### Group model building (GMB)

The workHORSE project workshops had the overall aim of involving stakeholders in the design process of the modelling tool and included a series of small-group exercises with specific objectives and outputs. Exercises were designed in the form of scripts [21]. We adapted previously validated scripts to our specific needs and context [22] based upon the work of Hovmand et al. [18] as part of a general framework; this allowed modelling teams to engage with stakeholders in the co-design of qualitative and quantitate models. Each script contained a succession of elements including descriptions of the exercise, purpose, time, materials needed, inputs, outputs, team roles required, steps and evaluation criteria. The use of scripts enabled better design of the workshops and more effective sessions leading to a more comprehensive and user-friendly workHORSE modelling tool and “buy in” from stakeholders.

### Data collection

Workshop 1 took place in February 2018 in Liverpool, UK. The team delivered two key activities. Activity 1 focused on developing a shared understanding of the NHSHCP and asked stakeholders to identify aspects of the programme that were working well, not well and their future hopes for the programme. During activity 2, stakeholders were asked to identify the key features that the workHORSE modelling tool should include that would make the tool useful for the decision-making process. Each activity was completed individually, followed by both table and whole group discussions. Each table had a mix of local, regional and national stakeholders to stimulate discussion. Stakeholders provided written feedback on post-it notes, and the table and group discussions were tape recorded and summarised on flipchart paper.

### MoSCoW approach

The acronym MoSCoW stands for Must-haves, Should-haves, Could-haves, and Would-haves (see explanation further below). Activity 2 resulted in stakeholders providing many suggestions. The feasibility of incorporating all stakeholders’ suggestions was limited due to the two-year timeline of the project. Therefore, we used the MoSCoW approach to prioritise the suggestions made by the stakeholders in order to reach a common understanding of the importance of their proposals [23]. The prioritisation process is based on the following categories [24]:

- Must have: the suggestions are critical to the project and without these the project will fail.
- Should have: the suggestions are important but are not as time dependent as the suggestions in the ‘must have’ category.
- Could have: the suggestions are desirable but not necessary.
- Would have: the suggestions are least important to the project and can be either dropped or incorporated at a later stage. The ‘W’ in the MoSCoW approach stands for “won’t have” however, for the purpose of the project, we changed it to “would have, time permitting”.

The project team initially utilised the MoSCoW approach to categorise the suggestions provided by the stakeholders. The results were then presented to the stakeholders and discussed until a consensus was reached.

### Framework analysis

The data (feedback on post-it notes, table and whole group discussion recordings and transcripts) was analysed using framework analysis [25]. The researcher (LH) consulted with the team during the different stages of the analysis. Step one involved data familiarisation. Once the researcher was immersed in the data, emerging themes were identified. After this a thematic framework was developed and refined by allowing the data to dictate the themes rather than by forcing the data to fit a priori themes or issues. During step three and four, the data corresponding to the different themes within the thematic framework were identified (indexing) and arranged in charts of the themes (charting) using line by line coding in NVivo. For the final step, mapping and interpretation, the data within the charts were analysed. During this process we included all categories of opinion by exploring, categorising and reporting all feedback. Data was triangulated by using feedback on post-it notes, table and whole group discussions, field notes of the research team and validating the model feature suggestions with stakeholders using the MoSCoW approach.

### Ethics

Ethical approval was granted by the University of Liverpool Health and Life Sciences Committee on Research Ethics (Psychology, Health and Society) on the 14th September 2017 (reference number 2242). Written consent was obtained from stakeholders prior to the workshop. All data was anonymised and stored in locked filing cabinets and on password protected computers.

## Results

Fifteen stakeholders accepted the invitation and participated in workshop 1. Stakeholders represented the local, regional and national perspective, with attendees from LAs, CCGs, GPs, Academia, Public Health England and third sector organisations. The verbatim quotes presented below are from different participants.

### NHS Health Checks Programme

Stakeholders identified two main aspects of the NHSHCP that are working well; first, the fact that it is a national programme for the identification of CVD, and second, that there is continued financial and political support for the programme.

*“So, I think the good thing is that it covers the whole population pretty much. Everybody has access to it and obviously you get identification of CVD.”* (Participant 11, regional).

*“Within LAs the funding is still there in terms of the public health budget. So, I suppose, because it’s got continued investment and there is disparity of course in the country obviously around how people are commissioning it, but I think within those, I think we are very lucky at the moment, we have quite a decent budget.”* (Participant 4, national).

However, many stakeholders highlighted issues that are not working so well within the NHSHCP. Even though some stakeholders noted an increase in coverage and uptake within their LA, variations exist in the uptake of NHS Health Checks. Overall, less than 50% of people have taken up their NHS Health Check invitation. Stakeholders suggested there is poor public understanding of NHS Health Checks and how they related to their own health due to suboptimal public engagement. In terms of the delivery of the NHSHCP, some stakeholders noted that General Practitioners are reluctant to deliver NHS Health Checks and that the quality of the NHS Health Check itself can be variable due to poor communication of risk to the patients or inadequate training of staff.

*“If I just look for what is not working so well from my point of view, I think that we all are, you know us as practitioners, we as people involved in the [NHS] Health Checks, we all know what we are talking about. When we go a few blocks down the road and we say can you have a [NHS] Health Check, they say what is that and why are we having these. So, it is saying why we are doing this and why it is important to get a [NHS] Health Check. Especially as we are looking at moving away from an NHS that treats ill health, to an NHS that prevents ill health. So, I think it is really important that we get that message [across].”* (Participant 11, regional).

*“There is no single approach to training staff. The competency framework does not hold anyone accountable.”* (Participant 10, local).

Depending on the outcome of the NHS Health Check, patients can be prescribed medication or referred to lifestyle services including smoking cessation or weight management services. Stakeholders emphasised the importance of what happens after the NHS Health Check rather than solely focusing on NHS Health Check completion. Lifestyle services are not mandated which means that some areas do not have the option to refer patients. However, where referral to lifestyle services was available, stakeholders reported referrals to be low and variable.

*“The [NHS] Health Check on its own is of limited value, ( …*) *actually what happens next which is the added value.”* (Participant 6, national).

*“( …*) *actually the [lifestyle] services that we refer into are not mandatory and they are changing as we re-commission. So, for instance, some areas are stopping smoking cessation, so there is a “so what” for me about this because it would be lovely to have a [NHS] Health Check but there is nothing there to refer them into. Where does that leave us?”* (Participant 3, local).

*“What is not working so well, and we need to work on are the very low referral rates into lifestyle interventions and very low prescription rates for people who have a [NHS] Health Check. [NHS] Health Checks don't seem to work, especially the lifestyle part. So, we need to work on that.”* (Participant 9, local).

Many stakeholders highlighted the issue of poor access to relevant and good quality data making it difficult to track individuals through the NHS Health Check process and subsequent lifestyle referrals. Accessibility to postcode data is also limited, thus leading to a lack of available information about whether deprived populations are being reached.

*“In terms of not working well kind of includes data and the lack of it and lack of join up and therefore lack of audit of outcomes.”* (Participant 4, national).

*“[We need to] be able to track people through the system, so we can try and understand real-time, how effective our model actually is, ultimately. Because we are quite blind at that at the moment.”* (Participant 2, local).

*“I can’t get postcode data, so I just know that X number of people in a GP practice have had a [NHS] Health Check. I don’t get to see the postcode data, so I don’t know about socio-economic status.”* (Participant 10, local).

Stakeholders also identified a lack of evidence base regarding the effectiveness and cost-effectiveness of the NHSHCP. Limited understanding of the potential cost savings to local governments for social care, benefits and sickness absence was also identified. Some stakeholders also mentioned an inadequate access to evidence-based interventions for the treatment of identified risk factors other than, for example, statins for high blood pressure.

*“Where is the evidence base? Where is the effectiveness? Where is cost-effectiveness?”* (Participant 7, national).

Some stakeholders noted a disconnect in the NHS Health Check system and reported a difference in the extent to which the programme is prioritised by politicians in local government (LA) and primary care. Furthermore, they believed that the cost of the NHS Health Check model is relatively high, while LA budgets are shrinking. There is some intelligence regarding the consideration of national guidelines in approaches within the NHSHCP, however it is not always clear where they feature and whether services are underpinned by best practice guidelines.

*“For me there is something about, the changing climate that we are working in and including the budgetary constraints that we are facing. So, there is less money to spend on [NHS] Health Checks.”* (Participant 12, regional).

*“It is unclear if the services that those receiving [NHS] Health Checks are referred onto are underpinned by best practice guidelines? Do those services exist to facilitate onward referral?”* (Participant 7, national).

Some stakeholders also commented that NHS Health Checks are based on outdated models. They noted that the NHS Health Check is based upon a bio-medical model that does not typically address the wider determinants of health. It was suggested a trans-theoretical model might better support and sustain interventions to improve health and wellbeing.

*“[NHS Health Checks are based on a] rather medicalised model. It doesn’t typically address wider determinants and is practically difficult to set up outside primary care.”* (Participant 3, local).

Although they identified several aspects of the NHSHCP that were not working well, stakeholders also expressed their future hopes for the programme, many of which addressed the concerns raised.

Future hopes included: 1) maximising coverage, uptake, and referrals, 2) producing additional evidence on population health, equity, and economic impacts, 3) expanding the programme beyond CVD outcomes (i.e. dementia and cancer), 4) improving support for clinical management of risk factors via guidelines or products to inform the NHS Health Check, 5) decreasing inequalities and improving health outcomes to become cost-effective / cost-saving, and 6) improved GP engagement in the NHSHCP via suitable trained and resourced practitioners.

***“****A more sophisticated evidence base for differential impact in wider and more complex population groups to inform recommendations and prioritisation at the end of a [NHS] Health Check, for example smoking vs BP control etc.”* (Participant 5, national).

*“That the NHS Health Check can be wider than simply cardiovascular outcome and include for example dementia or cancer.”* (Participant 13, national).

### Features to include in the modelling tool

Stakeholders identified many model features / specifications that should be included to make the workHORSE modelling tool useful in the decision-making process. These features / specifications are mainly a reflection of the issues identified and described in this paper regarding the perceived current limitations of the NHSHCP. Stakeholders stressed the importance of the ease of use of the tool and the need for the tool to be beta-tested within LAs to ensure this. Furthermore, the outputs must be audience-specific and fit-for-purpose including both simple and complicated outputs such as infographics and data in tables. The remaining key features / specifications related to the model inputs, outputs and scenarios.

*“This is a bit of a no-brainer and it’s really important because public health people we can be in our ivory towers and we like to have, to read 50-page evidence reports on things but actually what we want is a tool which is incredibly easy to use. ( …*) *Equally the outputs are in the same format, so you need to be able to press one button for people like our analysts ( …*), *who want things that are really complicated, lots of tables, lots of graphs and that, and then on the opposite extreme you want where you’ve got two sides of infographics that your council is going to understand so it might be a big ask and there’s lots of more complicated things but I think basically the simplicity of something to use and to understand the outputs is something that sort of stands out most to me.”* (Participant 12, regional).

#### Model inputs

In addition to identifying the need for relevant and good quality data, stakeholders emphasised that the data needed to be easily and routinely available, and preferably from a limited number of resources. Some stakeholders also provided suggestions for specific data inputs including eligible population (the Office for National Statistics or registered residents), mental health epidemiology, genomics data, and must account for different levels of motivation that patients may have to make lifestyle changes after the NHS Health Check.

*“It’s not just about outputs. How about inputs to make sure that what you ask for, is something people can actually get their hands on to put into the system, the model.”* (Participant 13, national).

*“So, it’s a consultation that we don’t use Office of National Statistics eligible population information but use registered residence as your denominator.”* (Participant 8, local).

#### Model outputs

Stakeholders would like the model outputs to include, a) different time frames, both short and long term, b) local outputs (including ward level), c) measurable improvements in population health (weight, smoking, drinking, and blood pressure), d) effectiveness and cost-effectiveness, and e) not just CVD outcomes.

*“I think for the model, it would be good to have simulation of impact and cost of short, medium and long term, kind of 5, 5-10 and more than 10 years because obviously we want to think long term but actually, the public health grant ends in 2-3 years. We also want to see some sort of short-term gain if there is any.”* (Participant 9, local).

*“It would be really good to have a model simulating impact at ward level.”* (Participant 9, local).

*“Not just CVD outcomes but include others like quality of life, dementia, respiratory and social care spending.”* (Participant 1, national).

#### Scenarios

Stakeholders expressed the need to model certain scenarios for the tool to be useful. Suggestions mainly focused on best delivery models for NHS Health Checks around improving uptake, interventions such as statins for high blood pressure and lifestyle interventions, outcomes and reducing health inequalities. Further suggestions included modelling the impact of 1) low versus high quality NHS Health Checks based upon staff competencies, 2) different providers delivering NHS Health Checks, 3) differential allocation of resources for NHS Health Checks for different groups, 4) clinical interventions, 5) cost of different implementation models (i.e. primary care versus community pharmacy) and, 6) local / structural interventions.

*“The ability to show what happens if using different forms of invite (letter, SMS, call) for different groups.”* (Participant 12, regional).

*“Does the impact model originally look at structural interventions, alongside the [NHS] Health Checks and the [NHS] Health Check would sort of focus that? I wonder if the sort of a micro cosmos that you can have structural interventions at a local authority level? So, things like tobacco declaration scenarios could be modelled like that. And I suppose to the different ways of delivering the [NHS] Health Checks, it could be a structural intervention alongside different levels of NHS Health Check provision. That structural interventions, which actually can be influenced or implemented by local commissioners for something realistic.”* (Participant 9, local).

#### Guidelines

From a national level perspective, issues were raised around the use of guidelines. Suggestions were made for the workHORSE modelling tool to refer to national guidelines to encourage and assist implementation drivers to action, and to frame the national context.

#### MoSCoW

The MoSCoW approach (Table 1) shows the categorised stakeholders’ suggestions for the features / specifications the workHORSE modelling tool should include to make it useful for their decision-making process. This categorisation was further discussed and refined with stakeholders during workshop 2.

**Table 1:** MoSCoW prioritization

Must	Should	Could	Would
• Expand beyond CVD, to other NCDs • Identify most effective / equitable NHS HC deliveries (areas for improvement) • Useful / flexible / adaptable ○ Typology of deliveries ○ Typology of preventative interventions • Local perspective • Evidence based	• Realistic data requirements • Model local policy interventions • User-defined simulation horizon • Include social care costs • Outputs tailored to target audience	• Simple user interface layout (with an “advanced” tab for expert users) • Identify best option for given budget • Reference to implementation and how to (practice guide) • Infographics	• Include mental health outcomes • Staff competencies (low vs. high quality NHS Health Check) • Include the Commissioning for Quality and Innovation framework • Ward level outputs • Genomics data

## Discussion

This study usefully captured stakeholders’ perspectives regarding the NHSHCP and the key features that the workHORSE modelling tool should include. There was continued financial and political support for the NHSHCP. However, many stakeholders highlighted issues concerning lack of data on processes and outcomes, variability in quality of delivery, and suboptimal public engagement. Stakeholders’ hopes included maximising coverage, uptake, and referrals, and producing additional evidence on population health, equity, and economic impacts. Key model suggestions focused on developing good-practice template scenarios, analysis of broader prevention activities at local level, use of accessible local data, broader economic perspectives, and fit-for-purpose outputs.

Stakeholders identified several issues including the lack of evidence base regarding the effectiveness, cost-effectiveness and equity of the NHSHCP. This is supported by a review conducted by Martin et al. [26] stating that the effect of the NHS Health Check on health outcomes is limited due to the paucity of robust evidence (from randomised controlled trials). There are matched studies available that use electronic primary care record databases to compare outcomes between attendees and non-attendees; however, the studies are limited due to a lack of standardised codes, clinical diagnoses, lifestyle factors and missing data. Furthermore, there is limited research on the impact of the NHS Health Check on lifestyle behaviours and referral to services. Related to the lack of evidence base is the issue of poor access to relevant and good quality data. Martin et al. [26] also reported ongoing issues with consistent recording of data related to the NHS Health Check including attendance and health outcomes. This makes it difficult to track people through the NHS Health Check process and subsequent lifestyle referrals. In addition, stakeholders expressed difficulties with accessing postcode data and therefore are unable to see whether deprived populations are being reached. These issues are extremely important and therefore it is necessary to improve data collection systems to ensure that the effectiveness, cost-effectiveness and equity of the programme can be accurately evaluated.

Stakeholders also identified a lack of public understanding with regards to how to participate in the NHSHCP. In addition, some stakeholders noted the variable / low uptake of NHS Health Checks. A recent systematic review conducted by Harte et al. [27] explored the reasons why people do not attend an NHS Health Check. They identified several main reasons including lack of awareness or understanding of the NHS Health Check. In addition, people were uncertain about the purpose of the NHS Health Check and the preventative nature of the programme. Finally, some people were aware of the programme and the preventative nature but chose not to attend as they preferred not to know their disease risk or did not want to be reminded of their unhealthy lifestyle. To increase the uptake of NHS Health Check it is important to address these issues and educate the public on the purpose and importance of attending an NHS Health Check.

The MoSCoW approach has proved valuable to the workHORSE project. We used the approach to successfully prioritise the features of the workHORSE modelling tool that are required in order to make the tool useful for the decision-making process. The MoSCoW approach has been designed for time-limited projects but has been mainly used in software development, project management and business analysis [24]. The current project has shown how MoSCoW has the potential to be used for these types of health service or public health research projects when engaging with stakeholders and designing a project or a tool.

This paper reports on the findings from workshop 1, the first in a series of four workshops with stakeholders to inform the features / specifications of the workHORSE modelling tool to assist with decision-making of potential alternate effective and equitable implementations of the NHSHCP. Development of computer models to inform decision-making tend to be developed with minimal or no consultation with potential end-users. However, the literature has identified the ‘evidence-policy gap’ whereby there is an apparent disconnect between the production of evidence and the use of evidence by policymakers [14]. Although models exist to allow collaborations between academics and policymakers, there is a difference between collaborative research practices, where each other’s skills and expertise are used for certain areas of the research process, and co-productive research practices, whereby engagement is done throughout the process, with equal control and decision-making [14]. The workHORSE project has implemented the co-production process by using GMB exercises, with continuous stakeholder engagement, via workshops and communication (email, in-person and our online platform the workHORSE eLab) between the workshops. This has enabled bridging the ‘evidence-policy gap’ and maximised the potential use of the final workHORSE modelling tool. Having a broad spectrum of stakeholders has been extremely valuable, providing a variety of perspectives and highlighting different requirements the workHORSE modelling tool should include.

### Strengths and limitations

To our knowledge, this is the first paper to explore and report upon the views of key stakeholders in relation to the NHSHCP. Stakeholders were provided with an opportunity to state what is working well, not so well and future hopes for the programme. Utilising co-production techniques together with adapted community-based modelling system dynamics has enabled the development of a fit-for-purpose decision-making tool, allowing the quantification of the potential effectiveness, cost-effectiveness and equity of the NHSHCP and alternative scenarios.

This study has some limitations. First, stakeholders were recruited through our networks, followed by using snowballing techniques if stakeholders were unable to attend. Although we attempted to reach a wide variety of stakeholders from different perspectives and organisations, due to issues of time and resource capacity, personnel from some organisations were unable to participate. Despite potential recruitment bias, we managed to recruit a variety of stakeholders from local, regional and national organisations with varying perspectives. We deliberately recruited a mixture of people whose prior viewpoint might be regarded as being ‘NHS Health Checks evangelists’ and ‘NHS Health Checks sceptics’ and people in between. Ultimately however, we believe the participants in the workshop were reasonably representative of the stakeholder population. Our approach could be improved in future projects by using stakeholder ID mapping [28].

Second, when stakeholders recorded their individual views and perspectives on post-it notes, at times stakeholders agreed with what another stakeholder had written and added their locality. While we were able to identify which participants said what by locality and managed to tease out the original participant, we were unable to extend any conclusions based on this as several participants had similar views but did not write this down individually. However, we believe we still report here rich information from a wide variety of stakeholders.

## Conclusions

We developed a shared understanding of the current implementation of the NHSHCP with stakeholders. The stakeholders demonstrated their commitment to NHSHCP whilst highlighting the perceived requirements for enhancing the service and discussing how the decision-making tool would be instrumental in this process. These suggestions for improvement informed subsequent workHORSE workshops, model development and will result in a co-produced open access decision-making planning tool at the end of the project.

## Abbreviations

BMA: British Medical Association
CCGs: Clinical Commissioning Groups
CVD: Cardiovascular disease
GMB: Group Model Building
GPs: General Practitioners
HC: Health Check
ICER: Incremental Cost-Effectiveness ratio
LAs: Local Authorities
NHSHCP: National Health Service Health Check Programme
NICE: National Institute for Health and Care Excellence
PHE: Public Health England
NCDs: Non-communicable diseases
SRQR: Standards for Reporting Qualitative Research
QALYs: Quality adjusted life years
UK: United Kingdom
